# Spray-Dried Potato Juice as a Potential Functional Food Component with Gastrointestinal Protective Effects

**DOI:** 10.3390/nu10020259

**Published:** 2018-02-24

**Authors:** Małgorzata Kujawska, Anna Olejnik, Grażyna Lewandowicz, Przemysław Kowalczewski, Renata Forjasz, Jadwiga Jodynis-Liebert

**Affiliations:** 1 Department of Toxicology, Poznan University of Medical Sciences, 30 Dojazd Str., 60-631 Poznań, Poland; liebert@ump.edu.pl; 2 Department of Biotechnology and Food Microbiology, Poznan University of Life Sciences, 48 Wojska Polskiego Str., 60-627 Poznań, Poland; aolejnik@up.poznan.pl (A.O.); gralew@up.poznan.pl (G.L.); 3 Institute of Food Technology of Plant Origin, Poznan University of Life Sciences, 31 Wojska Polskiego Str., 60-624 Poznań, Poland; przemyslaw.kowalczewski@up.poznan.pl; 4 Department of Pharmacology, Poznan University of Medical Sciences, 5a Rokietnicka Str., 60-806 Poznań, Poland; rforjasz@ump.edu.pl

**Keywords:** potato juice, anti-inflammatory activity, antiulcerogenic effect, gastrointestinal protection

## Abstract

Background: Peptic ulcer disease, including its complications and functional dyspepsia, are prevalent gastrointestinal diseases, etiopathogenesis of which is associated with mucosal inflammation. Research into new therapeutics capable of preventing or curing gastrointestinal mucosal damage has been steadily developing over past decades. This study was undertaken to evaluate whether a spray-dried preparation of potato juice is applicable for treating and preventing gastrointestinal mucosal damage. Methods: We assessed potential protective effects of spray-dried potato juice (SDPJ) against gut inflammation in the co-culture Caco-2/RAW264.7 system, as well as a gastroprotective activity in a rat model of gastric ulceration. Results: The obtained results indicated that SDPJ down-regulates lipopolysaccharide (LPS)-induced mRNA expression and protein production of proinflammatory cytokines IL-6 and TNF-α in the co-culture model. Moreover, SDPJ provided dose-dependent protection against LPS-induced disruption of intestinal barrier integrity. In rats, five-day pretreatment with SDPJ in doses of 200 mg/kg and 500 mg/kg suppressed HCl/ethanol-induced TNF-α expression in gastric mucosa by 52% and 35%, respectively. In addition, the pretreatment with the lower dose of SDPJ reduced the incidence of ulcers (by 34%) expressed as ulcer index. Conclusion: The spray-dried potato juice appears to be an attractive candidate for ameliorating inflammation-related diseases of the gastrointestinal tract.

## 1. Introduction

Peptic ulcer disease (PUD), including gastric and duodenal ulcers, is associated with defects in the gastrointestinal mucosa and is a prevalent gastrointestinal disease with high morbidity and mortality. The most common causes of PUD are *Helicobacter pylori* infection, as well as the use of anti-inflammatory drugs, which additionally aggravate the disease leading to complications, such as gastrointestinal haemorrhage or perforation. Since the use of nonsteroidal anti-inflammatory drugs and corticosteroids is becoming more widespread, peptic ulcer complications have emerged as a substantial healthcare problem [1,2]. In addition, infection by *H. pylori* causes chronic inflammation, which has been suggested to develop to gastric cancer—the third leading cause of cancer-related deaths, according to the World Health Organization (WHO) [3,4]. Interestingly, new evidence suggests that abnormalities in gastroduodenal mucosa are also present in a substantial group of patients suffering from functional dyspepsia [5]. Although eradication therapy for *H. pylori* is superior in PUD patients and some functional dyspepsia patients, pharmacological and nonpharmacological prevention and treatment to enhance the healing of the gastroduodenal region is highly recommended for patients from both groups [2,5]. In traditional European medicine, gastrointestinal disorders were commonly treated with freshly squeezed potato juice. Clinical trials have proved its potential for relieving dyspeptic complaints [6,7,8]. An increasing number of studies have revealed that potatoes, as a rich source of bioactive compounds, such as phenolics, proteins, glycoalkaloids (GAs), and lectins, exhibit health-promoting properties, including antioxidant, anti-inflammatory, and anticancer activity [9]. Generally, it is believed that the anti-inflammatory properties of potato are related to proteins with protease inhibitor activity. Potato proteins have been demonstrated to alleviate perianal inflammation by inhibiting faecal proteases in patients with gastrointestinal resections and in infants [10]. On the other hand, the anti-inflammatory effect of potato is suggested to be attributable to antioxidants, including phenolic acids, carotenoids, or anthocyanins [11]. Human cohort studies proved the systemic anti-inflammatory effect of potato as measured by serum C-reactive protein, at a level that was inversely correlated with the serum concentration of certain potato antioxidants [12,13]. The anticarcinogenic effects of potato have been attributed to the presence of anthocyanins, as well as GAs and lectins, which may be toxic in high doses. Nevertheless, a considerable amount of research has been devoted to the study of the inhibitory effects of potato GAs (solanine and chaconine) on the growth of human cancer cell lines including human colon and stomach cancer cells [9,13]. GAs have been demonstrated to reduce cancer metastasis by suppression of the phosphoinositide 3-kinase (PI3K)/Akt/NF-kB signalling pathway and to induce apoptosis through caspase-3 activation and inhibition of ERK 1/2 phosphorylation, and α-chaconine was proved to be more effective than α-solanine [9]. Although in vitro studies are burdened with limitations, their findings suggest that potato GAs may not be as menacing as once thought [13].

The nutritional approach is recommended for complementary treatment of gastrointestinal diseases, including peptic ulcer, to prevent, alleviate or even heal the symptoms involving this pathology [14]. The application of freshly-squeezed potato juice is, however, troublesome, as it is extremely unstable. It instantly undergoes colonisation with microorganisms and changes its physicochemical, sensory, and nutritional properties. Therefore, we have developed a formula for potato juice for its using in health-oriented food products. Previously, we designed functional food products, such as pâtés, pasta, and frankfurters fortified with potato juice, addressed to patients with inflammatory bowel disease [15,16,17]. The object of the present study is spray-dried potato juice (SDPJ), which is suitable for incorporation into food products and dietary supplements. As heat processing can change phytochemical content in potato juice, as well as its bioactivity, our aim here was to evaluate whether SDPJ is capable of ameliorating inflammation-related diseases of the gastrointestinal tract.

## 2. Materials and Methods

### 2.1. Test Material

Fresh potato juice was collected as a by-product of potato starch production from a starch plant (WPPZ S.A., Luboń, Poland) according to the HACCP (Hazard Analysis and Critical Control Point) principles. Potato varieties with white and yellow flesh were derived from Polish cultivated areas. The juice was cooled to 4 °C and transported to our laboratory, where it was subjected to a spray drying process according to the procedure described previously [17,18]. Drying was carried out in a pilot-scale P-dryer Niro Atomizer 6.3 (GEA Co., Soeborg, Denmark) using the following conditions: 170 °C at the inlet to the drying chamber, 95 °C at the outlet, and the feed rate of juice 12 L/h.

### 2.2. Spray-Dried Potato Juice Analysis

Protein content in SDPJ was determined by Kjeldahl’s method in accordance with the 1871:2009 standard method, and the ash content according to ISO 763:2003.

Analyses of the SDPJ phenolic compounds were performed using an Agilent 1200 series HPLC system (Agilent Technologies, Inc., Santa Clara, CA, USA) equipped with a G1312A binary pump, a G1329A autosampler, a G1316A temperature-controlled column compartment, and a G1315D photodiode array detector. Chromatographic separations were carried out on an Agilent ZORBAX SB-C18 column at 25 °C. The mobile phase consisted of two solvents: 5% (*v*/*v*) formic acid in water (A) and methanol (B). A gradient elution procedure was performed as follows: 5%−20% B, 0−10 min; 20% B, 10−15 min; 20%–30% B, 15–30 min; 30% B, 30–35 min; 30%–45%, 35–50 min; 45%, 50–60 min; and 45%−5% B, 60–70 min. The flow rate was at 1 mL/min, and the injection volume was 10 μL. The HPLC chromatograms were recorded at 280 and 320 nm. Quantification of SDPJ phenolic compounds was based on the standards of 5-*O*-caffeoylquinic (chlorogenic), ferulic, and caffeic acids. Data acquisition and processing were performed using an Agilent ChemStation for LC 3D Systems software rev. B.03.02 (Agilent, Santa Clara, CA, USA).

The β-carotene content was determined by UV-VIS (ultraviolet-visible) spectrophotometric method according to Biswas et al., 2011 [19].

The concentration of GAs (α-chaconine and α-solanine) in SDPJ was determined using an isocratic HPLC method with ultraviolet detection at 200 nm [20].

The total antioxidative activity was determined by ABTS radical method (2,2′-azinobis-(3-ethylbenzothiazoline-6-sulfonic acid)) according to Re et al. [21] and expressed as mmol of Trolox equivalents per 100 g of dry matter (DM).

The total content of phenolic compounds was assessed using Folin–Ciocalteu reagent [22] and expressed as an equivalent of the chlorogenic acid (CAE) per 100 g of DM.

### 2.3. In Vitro Experiments

#### 2.3.1. Cell Cultures

A co-culture model consisting of a differentiated 21-day Caco-2 cell monolayer and a 24-h culture of RAW264.7 macrophages (both obtained from the European Collection of Cell Cultures and supplied by Sigma-Aldrich, St. Louis, MO, USA) was used to determine the anti-inflammatory effects of SDPJ. In this co-culture system, the RAW264.7 macrophages were stimulated with lipopolysaccharides to induce an inflammatory response, while the Caco-2 cells were exposed to SDPJ at concentrations of 0.01, 0.1, and 1 mg/mL, and budesonide (a reference glucocorticoid with strong anti-inflammatory potential). The anti-inflammatory activity of the test material was determined as described previously [23].

#### 2.3.2. Cytotoxicity Assay

To detect the cytotoxic effect of SDPJ, lipopolysaccharides (LPS), and budesonide on the Caco-2 and RAW264.7 cells under experimental conditions, the release of lactate dehydrogenase (LDH) was determined using a CytoTox-One™ Homogeneous Membrane Integrity Assay, according to the manufacturer’s protocol (Promega GmbH, Mannheim, Germany).

#### 2.3.3. Transepithelial Electrical Resistance

The integrity of the Caco-2 monolayer was assessed by measuring the transepithelial electrical resistance (TEER) using the Millicell Electrical Resistance System (Millipore, Merck KGaA, Darmstadt, Germany).

#### 2.3.4. Quantification of Proinflammatory Gene Expression Using Real-Time PCR

Total RNA was isolated from the RAW264.7 macrophages using TRI-Reagent (Sigma-Aldrich) according to the manufacturer’s instructions. The total RNA was reverse-transcribed using a Transcriptor First Strand cDNA Synthesis kit (Roche Diagnostics GmbH, Mannheim, Germany), following the manufacturer’s protocol. The resulting cDNA was amplified using a real-time quantitative PCR (polymerase chain reaction) system (SmartCycler DX real-time PCR system, Cepheid, Sunnyvale, CA, USA) with SYBR Select Master Mix (Life Technologies, Carlsbad, CA, USA). The following primers were used: β-actin forward primer: 5′-ATGG AGGG GAAT ACAG CCC-3′; β-actin reverse primer: 5′-TTCT TTGC AGCT CCTT CGTT-3′; IL-6 forward primer: 5′-TCTG AAGG ACTC TGGC TTTG-3′; IL-6 reverse primer: 5′-GATG GATG CTAC CAA ACT GGA-3′; TNF-α forward primer: 5′-AGGG TCTG GGCC ATAG AACT-3′; and TNF-α reverse primer: 5′-CCAC CACG CTCT TCTG TCTAC-3′. The level of transcripts was normalized using β-actin as an internal standard. Quantitative gene expression analysis was carried out following the protocol described previously [23].

#### 2.3.5. Determination of TNF-α and IL-6

The secretion of TNF-α and IL-6 cytokines was determined in LPS-stimulated RAW264.7 macrophages using enzyme-linked immunosorbent assay (ELISA) kits (R&D Systems Inc., Minneapolis, MN, USA) according to the manufacturer’s instructions.

### 2.4. In Vivo Experiment

#### 2.4.1. Experimental Design

Animals: We used male Wistar rats bred in the Department of Toxicology, Poznan University of Medical Sciences (Poznań, Poland). Animals were held (4 rats/cage) in polycarbonate cages (Techniplast, Italy) with wood shavings at 22 ± 2 °C, 40%–54% humidity, and controlled circulation of air with a 12 h light/dark cycle. A commercial diet (ISO 22000 certified laboratory feed Labofeed H) and drinking water were available ad libitum.

Experimental design: Sixty (12-week old) rats weighing 250 ± 22 g were divided randomly into five groups, twelve animals in each. The experiment was performed according to the procedure described by Caldas et al. [24] with slight modifications. Three groups of rats were orally treated with the suspension of the preparation of dried potato juice in water in a dose of 500 mg/kg b.w. per day (groups II and V) and 200 mg/kg b.w. per day (group IV) for five days. On the fifth day of the experiment one hour after SDPJ treatment, animals from groups III–V orally received the mixture of 0.3 M HCl and 60% ethanol (1:1) in a dose 1 mL per 150 g b.w to induce acute gastric lesions. Group I (control) and group II were given distilled water. The animals were sacrificed by decapitation 2 h after induction of gastric lesions; their stomachs were removed and examined for quantification of the lesions. Photographs of haemorrhagic erosions were taken, and specimens of gastric mucosa were collected.

Before starting the in vivo study, we had conducted a 90-day subchronic toxicity test on rats (data not shown), the results of which confirmed the safety of SDPJ at both doses used in the experiment. This animal study followed the animal welfare regulations according to EU Directive 201/63/EU, and it was approved by the Local Animal Ethics Committee for Animal Experimentation (protocol No. 52/2012).

#### 2.4.2. Ulcer Index

The number and severity of haemorrhagic lesions per stomach were scored according to the following scoring system: 0 = no pathology; 1 = a small ulcer (1–2 mm); 2 = a medium ulcer (3–4 mm); 4 = a large ulcer (5–6 mm); and 8 = a larger ulcer (>6 mm). The mean ulcer index UI ± SD (standard deviation) was expressed as the sum of the total scores divided by the number of animals in a group [25].

#### 2.4.3. Inflammatory Cytokines (TNF-α and IL-6)

Levels of TNF-α and IL-6 were quantified by using ELISA kits according to the manufacturer’s instructions (R&D Systems, Inc. Minneapolis, MN, USA). Gastric mucosa tissue homogenate was prepared with nine volumes of 50 mM phosphate buffer, pH 7.4.

### 2.5. Statistical Analysis

All analyses were performed with the use of the GraphPad InStat statistical package, version 3, (GraphPad Software Inc., San Diego, CA). The mean values and standard deviations were calculated. One-way analysis of variance (ANOVA) followed by the Student-Newman-Keuls test for multiple comparisons was used, *p* < 0.05 was considered the limit of significance.

## 3. Results

### 3.1. Spray-Dried Potato Juice Characteristics

Results of the determination of bioactive compounds in SDPJ are presented in Table 1.

### 3.2. Effect of Spray-Dried Potato Juice on Intestinal and Macrophage Cell Viability

**Figure 1:**
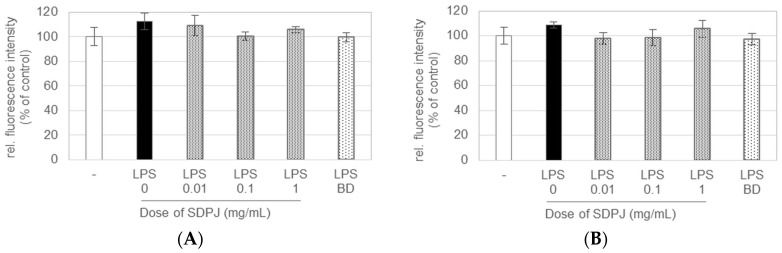
Effect of spray-dried potato juice (SDPJ) and budesonide (BD) on Caco-2 cells (**A**) and RAW264.7 macrophage (**B**) viability determined in a Caco-2/RAW264.7 co-culture system nonstimulated (−) or stimulated with lipopolysaccharides (LPS). Values represent the means ± SD (*n* = 3).

**Figure 2:**
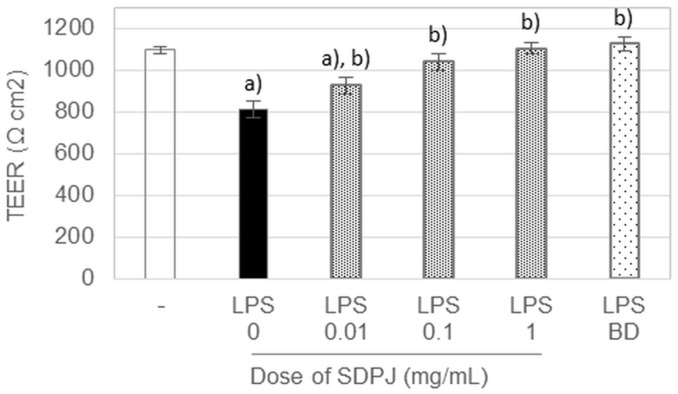
Effect of spray-dried potato juice (SDPJ) and budesonide (BD) on TEER values determined in a co-culture system of Caco-2 cells and RAW264.7 macrophages nonstimulated (−) or stimulated by lipopolysaccharides (LPS). Values represent the means ± SD (*n* = 3). a) *p* < 0.05 vs the untreated group. b) *p* < 0.05 vs the LPS-treated group.

Prior to the assessment of the ability of SDPJ to suppress the LPS-induced production of inflammatory cytokines, an LDH assay was performed to exclude the possibility of an inhibitory effect caused by cytotoxicity. The viability of both Caco-2 cells treated with SDPJ and LPS-activated RAW264.7 macrophages did not differ significantly (*p* > 0.05) from the untreated cells constituting the control cell culture system. The analysed preparation of potato juice at concentrations ranging from 0.01 to 1.0 mg/mL did not show any cytotoxic effects on differentiated intestinal Caco-2 cells (Figure 1A) and RAW264.7 macrophages (Figure 1B), respectively. Moreover, the treatment of the non-activated Caco-2/RAW264.7 model with SDPJ at doses of 0.01, 0.1 and 1.0 mg/mL did not affect either the intestinal barrier integrity, or the transepithelial permeability (Figure 2).

### 3.3. Effects of Spray-Dried Potato Juice on TEER in the Caco-2/RAW264.7 Co-Culture System

The stimulation of RAW264.7 cells with LPS was followed by a 26% decrease in TEER, which serves as a marker of the Caco-2 monolayer integrity. Treatment with budesonide or SDPJ at all tested doses protected the Caco-2 cell monolayer against injury induced by LPS. Furthermore, the TEER values measured in LPS-stimulated cells treated with the tested preparation of potato juice at doses of 0.1 and 1.0 mg/mL were quantitatively comparable to that of budesonide and were similar to that determined in the control cells, which were not treated with LPS (Figure 2).

### 3.4. Anti-Inflammatory Effects of Spray-Dried Potato Juice in the Caco-2/RAW264.7 Co-Culture System

**Figure 3:**
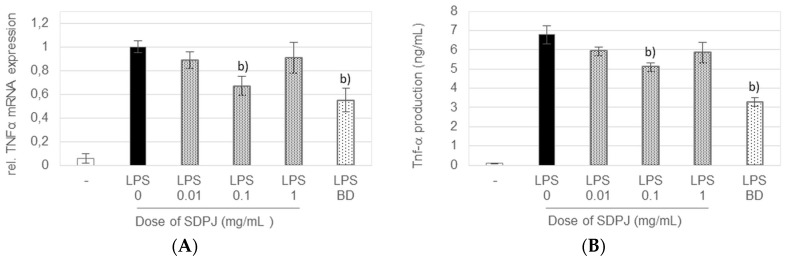
Effect of spray-dried potato juice (SDPJ) and budesonide (BD) on mRNA expression of TNF-α (**A**) and the production of TNF-α (**B**) in a co-culture system of Caco-2 cells and RAW264.7 macrophages nonstimulated (−) or stimulated by LPS. Values represent the means ± SD (*n* = 3). b) *p* < 0.05 vs. the LPS-treated group.

**Figure 4:**
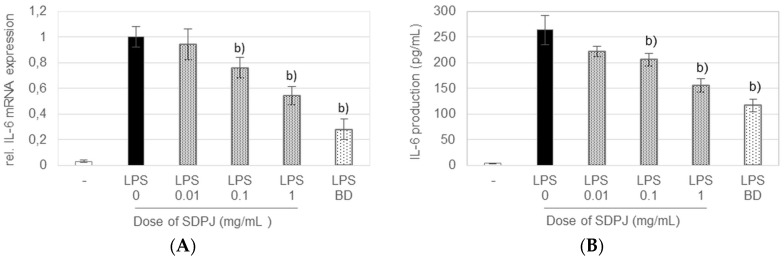
Effect of spray-dried potato juice (SDPJ) and budesonide (BD) on mRNA expression of IL-6 (**A**) and the production of IL-6 (**B**) in a co-culture system of Caco-2 cells and RAW264.7 macrophages nonstimulated (−) or stimulated by LPS. Values represent the means ± SD (*n* = 3). b) *p* < 0.05 vs. the LPS-treated group.

Stimulation of the RAW264.7 cells with LPS significantly upregulated the mRNA expression and production of both TNF-α and IL-6 (Figure 3 and Figure 4). The LPS-induced overexpression of TNF-α at both mRNA and protein level was significantly suppressed by the treatment with the tested preparation of potato juice at a dose of 0.1 mg/mL, by 33 and 25%, respectively (Figure 3).

SDPJ at doses of 0.1 and 1.0 mg/mL also caused a remarkable dose-dependent decrease in the induced IL-6 mRNA level, by 24 and 46%, respectively (Figure 4A), and in IL-6 secretion by 22 and 41%, respectively (Figure 4B). However, the inhibitory effect of SDPJ on the induced gene expression and secretion of both cytokines was lower than that of budesonide.

### 3.5. Anti-Ulcerogenic Effect of Spray-Dried Potato Juice in Rats

**Figure 5:**
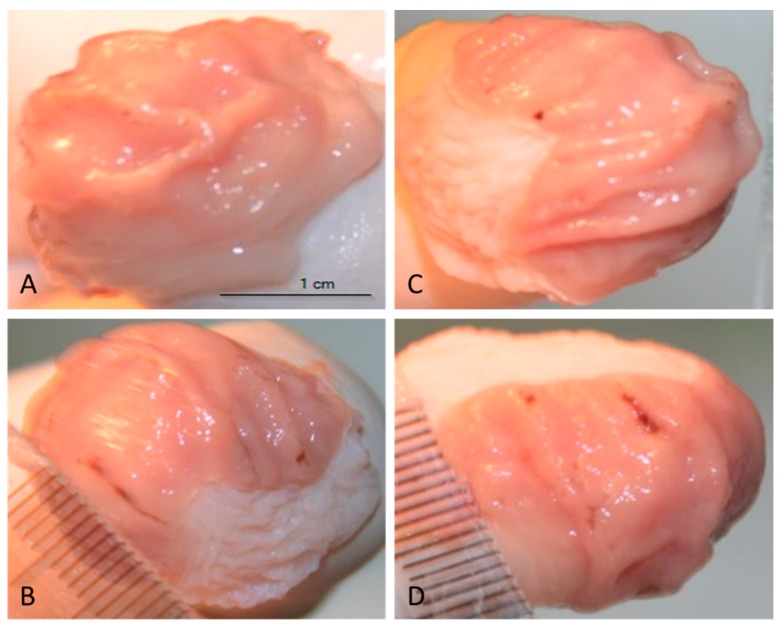
Representative images of gastric lesions in rats treated with spray-dried potato juice (SDPJ) (**A**) SDPJ 500 mg/kg alone; (**B**) rats treated with the mixture of HCl/ethanol alone; (**C**) SDPJ 200 mg/kg + HCl/ethanol; and (**D**) SDPJ 500 mg/kg + HCl/ethanol.

As shown in Figure 5B, administration the mixture of HCl/ethanol induced extensive visible haemorrhagic red bands of different sizes along the stomach. Rats pretreated with the lower dose of SDPJ (200 mg/kg b.w.) before the administration of the mixture of HCl/ethanol had considerably fewer regions of gastric ulcer development (Figure 5C) compared with the rats administered the mixture alone (Figure 5B). The ulcer index calculated from the size and number of the gastric lesions was 29 ± 10 in HCl/ethanol-treated rats. SDPJ treatment at a dose of 200 mg/kg significantly inhibited the formation of gastric lesions (ulcer index = 19 ± 12), while a dose of 500 mg/kg did not affect the HCl/ethanol-induced gastric ulceration (ulcer index = 25 ± 15) (Figure 5D).

### 3.6. Anti-Inflammatory Effects of Spray-Dried Potato Juice in Rats

**Figure 6:**
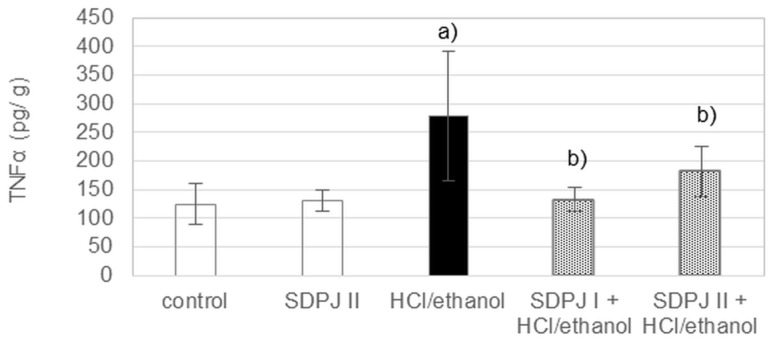
Effect of spray-dried potato juice (SDPJ) on level of gastric TNF-α in rats treated with the mixture of HCl/ethanol. SDPJ I: SDPJ 200 mg/kg. SDPJ II: SDPJ 500 mg/kg. HCl/ethanol: the mixture of 0.3 M HCl and 60% ethanol (1:1). Values represent the means ± SD (*n* = 8). a) *p* < 0.05 vs. the untreated group. b) *p* < 0.05 vs. the HCl/ethanol-treated group.

Treatment of rats with the mixture of HCl/ethanol caused a significant increase in the gastric mucosal level of TNF-α by 123%, as compared to controls. The tested preparation of potato juice significantly inhibited the induced secretion of TNF-α in rats administered 200 and 500 mg/kg b.w., by 52% and 35%, respectively, as compared to the ulcer-induced rats (Figure 6).

**Figure 7:**
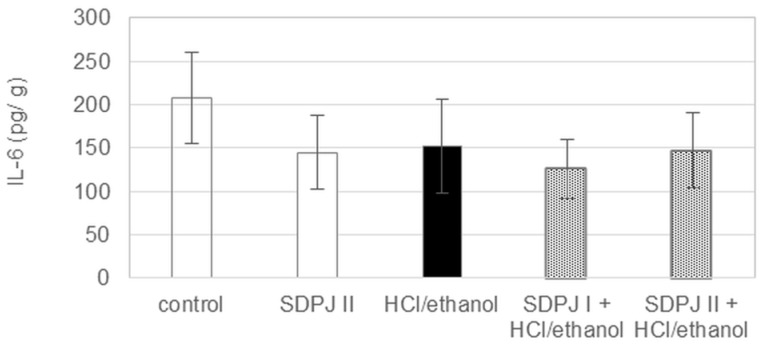
Effect of spray-dried potato juice (SDPJ) on the level of gastric IL-6 in rats treated with the mixture of HCl/ethanol. SDPJ I: SDPJ 200 mg/kg. SDPJ II: SDPJ 500 mg/kg. HCl/ethanol: the mixture of 0.3 M HCl and 60% ethanol (1:1). Values represent the means ±SD (*n* = 8).

In this experiment, IL-6 level was not affected significantly neither by the mixture of HCl/ethanol, nor SDPJ (Figure 7).

## 4. Discussion

A recent study demonstrated that *Helicobacter pylori* impairs the defensive response of gastric epithelial cells to acid, contributing to diminished barrier function and inflammatory response, and consequently leading to mucosal injury [3]. Although the pathogenesis of functional dyspepsia (FD) is not fully understood, some studies have provided evidence for the presence of minimal inflammation in the duodenal mucosa of the FD patients. It has been hypothesised that increased intestinal permeability is a potential pathogenic mechanism that could be involved in the generation of low-grade duodenal inflammation and consequently symptoms of FD [26]. Potato antioxidants such as phenolic acids, mainly chlorogenic, caffeic and ferulic acids, and carotenoids as well as GAs have been reported to be capable of lowering inflammation and might be useful in treating inflammatory disease conditions including gastrointestinal disorders [13]. However, the content of phenolic compounds in potato tubers has been found to vary depending on the variety [9]. To examine whether spray-dried potato juice can protect gastrointestinal mucosa against inflammatory damage we assessed its effect on intestinal barrier integrity and production of proinflammatory mediators. The effect of SDPJ on barrier dysfunction was investigated by measuring TEER in an intestinal epithelial Caco-2 cells/macrophage RAW264.7 cells co-culture system. The RAW264.7 cells were stimulated with bacteria-derived LPS to induce the inflammatory response, while the Caco-2 cells were exposed to SDPJ at doses of 0.01, 0.1, and 1.0 mg/mL. The treatment with the tested preparation of potato juice provided a dose-dependent protection against LPS-induced disruption of monolayer integrity, with complete protection occurring at 1.0 mg/mL. It seems probable that this effect is attributed to chlorogenic acid—the main polyphenol found in yellow and white potatoes—since it has been demonstrated to be mostly taken up by the Caco-2 cells [27]. In this context, our suggestion is in agreement with Ruan et al. [28] who have found that chlorogenic acid decreased intestinal permeability by the maintenance of tight junction protein expression, including zonula occludens-1 and occludin, in LPS-challenged rats. Whether increased permeability is the cause, or the consequence of low-grade inflammation has not yet been fully determined, notwithstanding a close relationship between impaired integrity and inflammatory activity has been proven both in in vitro and in vivo studies [26,28]. In chronic inflammation, macrophages have been reported to exert an important role locally by releasing cytokines including TNF-α and IL-6 and, therefore, inhibition of these inflammatory agents is expected to reduce or inhibit disease progression [29]. Hence, in this experiment, we evaluated the influence of SDPJ on the secretion of proinflammatory cytokines in cell culture. The treatment of RAW264.7 cells with the tested preparation of potato juice decreased the LPS-induced expression of mRNA for TNF-α and IL-6 which was accompanied by a decrease in their secretion. Recently, similar inhibitory effects on the production of the proinflammatory cytokines in LPS-induced RAW 264.7 cells have been demonstrated for purple sweet potato extract [30], as well as for potato peel extracts enriched in individual potato GAs [31], and different potato phytochemicals have been shown to contribute to this effect. Sugata and co-workers [30] have reported that antioxidative compounds, such as phenolic acids, flavonoids, and anthocyanins, suppressed the production of nitric oxide, and some proinflammatory mediators and cytokines, including NFκ-β, TNF-α, and IL-6. The beneficial effect of polyphenols on barrier integrity associated with inhibition of inflammatory response has been reported by Ruan et al. [28]. The authors have found that chlorogenic acid decreased the level of IFN-γ and TNF-α in the jejunum and colon in LPS-challenged rats leading to improved intestinal permeability. Surprisingly, Kenny et al. [31] have found that potato GAs in sub-toxic concentrations exerted anti-inflammatory activity in vitro. The authors suggested that this property may be associated with structural similarities of their aglycones to diosgenin, a precursor of steroidal hormones, and anti-inflammatory steroids [31].

We verified via an animal experiment the gastroprotective activity of the dried potato juice, demonstrated in vitro. However, some concerns arise from the probable presence of GAs, primarily, solanine, and chaconine, in potato juice [32]. It has been reported that improper harvest and storage methods may result in the formation of GAs in potatoes. Established acceptable limit of GA content in potatoes is 200 mg/kg of fresh potato tubers [33]. The tested dried potato juice contained 591.2 ± 11.4 μg of α-solanine/g DM and 990.1 ± 19.9 μg of α-chaconine/g DM, and their contents, in terms of fresh weight, did not exceed the limit values. The doses of SDPJ used in the in vivo experiment were chosen based on available literature and did not cause any toxic effects related to the presence of GAs [33]. The potential gastroprotective activity of the tested preparation of potato juice was evaluated in rats using necrotising agents, such as ethanol and HCl, which have been demonstrated to induce the gastric mucosal injury and gastritis. The mucosa injury has been demonstrated as haemorrhagic red bands of different size along the glandular stomach [34]. The pre-treatment with the lower dose of SDPJ (200 mg/kg b.w.) effectively reduced the severity of gastric damage and incidence of ulcers. This is the first study demonstrating the anti-ulcerogenic activity of potato juice subjected to a spray-drying process [18]. Ethanol causes acute inflammatory responses as a part of a defence mechanism against tissue damage attributed to an imbalance between proinflammatory cytokines, including TNF-α and IL-6, and anti-inflammatory cytokines (e.g., IL-10) [26]. In this experiment, the tested preparation of potato juice at both tested doses significantly inhibited ethanol-induced secretion of TNF-α in the gastric mucosa. For IL-6, we observed a tendency to reduce its level; however, the difference was not significant. The results demonstrated that pretreatment with SDPJ at a dose of 200 mg/kg b.w. notably inhibited HCl/ethanol-induced gastric lesions by inhibiting inflammatory reactions. This agrees with previous reports demonstrating the ability of products made from potatoes to relieve inflammation in vivo [35,36,37]. It has been reported that potato peel extract can inhibit the production of pro-inflammatory mediators, such as nitric oxide, prostaglandin E2 (PGE2), TNF-α, and IL-6, and expression of inducible nitric oxide synthase and cyclooxygenase-2 (COX-2) protein in colonic tissue of mice with dextran sulfate sodium (DSS)-induced colitis, resulting in the prevention of colitis development [35]. Potato extract has been shown to increase the expression of IL-10 and reduce the expression of TNF-α in serum and lung tissues of rats with cigarette smoke-induced chronic obstructive pulmonary disease [36]. In mice with chemically-induced atopic dermatitis, potato extract alleviated the exacerbation of skin lesions by suppressing total serum level of IgE and maintaining T helper 1 (Th1; interferon-γ, and IL-12) and Th2 cytokines (IL-4 and IL-13) balance [37]. In addition, there are several reports on beneficial effects on colitis of pure phenolic acids present in potato juice. Ferulic acid, as well as chlorogenic and caffeic acid, have been demonstrated to ameliorate trinitrobenzene sulfonic acid and DSS-induced ulcerative colitis in rodents, respectively, which was accompanied by down-regulation of synthesis of proinflammatory cytokines, including IL-1β [38,39], TNF-α, IL-6, and COX-2 [38] in the colon. There is also clinical evidence that consumption of yellow potato, rich in carotenoids, can decrease inflammation associated with IL-6 production [11].

Results of our study demonstrate that some parameters, i.e., antiulcer effect and TNF-α gastric level display a biphasic dose-response. This type of response is defined as “a low-dose stimulation and high-dose inhibition” [40]. The size and number of gastric lesions were substantially reduced in rats pretreated with the lower dose of SDPJ; however, the higher dose did not enhance this effect. Simultaneously, the decrease in the level of TNF-α was more pronounced in rats administered the lower dose of SDPJ, which was in line with the in vitro findings presented above. Data on this phenomenon in animal experiments are much less abundant than in cell culture assays. One of the examples is a report of Dey et al. [41], who demonstrated the protective effect of resveratrol on an indomethacin-induced gastric ulcer in mice was exerted by a dose 2 mg/kg, whereas the effect of a 10 mg/kg dose was the opposite. The authors suggested that the explanation could be the inhibition of cyclooxygenase-1 by resveratrol, which reduced PGE2 synthesis and angiogenesis, leading to delayed healing. On the basis of the results obtained, it is difficult to explain the reason for the observed biphasic dose-response. However, it could be speculated that multiple bioactive components present in the tested preparation (SDPJ) might disturb at high doses the process of healing by various mechanisms, including modulation of some cytokines production.

Increasing evidence suggests that the anti-inflammatory potential of functional foods is attributed to the synergy of bioactive compounds as well as to the specific interactions with other nutrients. As there are many compounds present in the tested preparation of potato juice, such as phenolic acids, glycoalkaloids, and proteins, which have been suggested to contribute to gastrointestinal protection, further studies on their biological activities could improve our knowledge about the mechanisms underlying the beneficial effects observed in the current experiments.

## 5. Conclusions

In conclusion, the presented findings demonstrate that spray-dried potato juice remarkably suppresses inflammatory response in vitro in activated macrophages and in the gastric mucosa of experimentally induced gastric lesions in rats. In addition, the tested preparation was beneficial for relieving the severity of gastric ulcers in rats. Hence, the potato juice, processed by spray-drying, appears to be an attractive candidate for ameliorating inflammation-related diseases of the gastrointestinal tract, including peptic ulcer disease and functional dyspepsia. Moreover, its implementation is feasible, since the spray-dried formulation is suitable for the incorporation of potato juice into food products and dietary supplements

## Figures and Tables

**Table 1:** Content of bioactive compounds, as well as antioxidant activity of SDPJ.

Parameter	Unit	Amount
Dry matter	g/100 g FM	90.1 ± 0.5
Protein	g/100 g DM	49.22 ± 0.40
Ash	g/100 g DM	16.34 ± 0.09
**Total phenolic compounds:**		
by Folin-Ciocalteu	mg CAE/100g DM	366 ± 35
by HPLC	mg CAE/100g DM	330.2 ± 12.0
**Individual phenolic compound:**		
Chlorogenic acid	mg/100g DM	13.3 ± 1.8
Ferulic acid	mg/100g DM	17.8 ± 2.3
Caffeic acid	mg/100g DM	22.2 ± 1.4
β-carotene	mg/100g DM	0.020 ± 0.008
**Glycoalkaloids:**		
α-solanine	mg/100 g DM	59.1 ± 1.1
α-chaconine	mg/100g DM	99.0 ± 2.0
Antioxidant activity *	mmol TEAC/100g DM	26 ± 2

SDPJ: spray-dried potato juice. FM: fresh matter. DM: dry matter. CAE: chlorogenic acid equivalent. TEAC: Trolox equivalent antioxidant capacity. * According to ABTS method
